# Solar-driven S-scheme magnetic CoFe_2_O_4_/CdO@bentonite heterostructure for concurrent Cr(VI) reduction and cefixime degradation: mechanistic insights, kinetics, and real wastewater validation

**DOI:** 10.1038/s41598-026-53606-0

**Published:** 2026-05-28

**Authors:** Moones Honarmand, Faramarz Sanandaji, Ahmad Aryafar

**Affiliations:** 1 Department of Chemical Engineering, Birjand University of Technology, Birjand, Iran; 2 Department of Mining Engineering, Faculty of Engineering, University of Birjand, Birjand, Iran

**Keywords:** Cobalt ferrite, Cadmium oxide, Bentonite, Heterojunction, Photocatalysis, Magnetic recovery, Chemistry, Engineering, Environmental sciences, Materials science

## Abstract

A novel solar-responsive magnetic CoFe_2_O_4_/CdO@bentonite (CF-CdO@B) heterostructure was rationally engineered. The incorporation of bentonite as a support matrix enabled improved dispersion, enhanced interfacial contact, and increased adsorption capacity, while the integration of CoFe_2_O_4_ and CdO facilitated efficient charge separation. Comprehensive structural, optical, and electrochemical characterizations confirmed the successful formation of a tightly coupled heterointerface. Under sunlight irradiation, the optimized photocatalyst achieved rapid and complete removal of Cr(VI) and cefixime within 25 and 60 min, respectively. Kinetic analysis revealed pseudo-first-order behavior with significantly enhanced rate constants (0.6447 min^-1^ for Cr(VI) and 0.2686 min^-1^ for cefixime), outperforming the individual components by an order of magnitude. Mechanistic investigations demonstrated that the superior photocatalytic activity originates from an S-scheme charge transfer pathway. The catalyst exhibited excellent magnetic recoverability, structural stability, and reusability over multiple cycles. Importantly, its practical applicability was validated in complex real water matrices, achieving high cefixime removal efficiencies (94% in tap water, 91% in river water, and 85% in hospital wastewater) and complete Cr(VI) reduction even in industrial effluents. This study provides a robust and sustainable strategy for dual-function photocatalysis, offering new insights into S-scheme heterojunction design and highlighting the potential of CF-CdO@B as an efficient candidate for real-world wastewater remediation.

## Introduction

The rapid expansion of industrial activities and pharmaceutical consumption has led to the continuous release of complex mixtures of inorganic and organic contaminants into aquatic environments. Hexavalent chromium [Cr(VI)], a highly toxic and carcinogenic heavy metal, and antibiotics such as cefixime are two important classes of pollutants that frequently coexist in industrial and municipal effluents. Their persistence and distinct chemical behavior require treatment strategies capable of simultaneously enabling both reductive and oxidative transformations, which remains a major challenge for conventional wastewater treatment technologies^1,2^.

Among advanced remediation approaches, solar-driven photocatalysis has attracted increasing attention due to its sustainability and ability to utilize renewable energy^3,4^. However, the performance of single-component photocatalysts is often limited by poor visible-light absorption, rapid recombination of photogenerated charge carriers, and insufficient redox ability for treating diverse pollutants. Therefore, rational material design, particularly heterojunction engineering, has emerged as an effective strategy to overcome these limitations^5,6^.

Cobalt ferrite (CoFe_2_O_4_), a spinel ferrite semiconductor, has been widely investigated in photocatalysis due to its narrow band gap, chemical stability, and intrinsic magnetic properties, which enable easy catalyst recovery^7,8^. However, its photocatalytic efficiency is still limited by moderate charge separation and rapid recombination of photogenerated electron–hole pairs, which reduces the formation of reactive oxygen species. Similarly, cadmium oxide (CdO), an n-type semiconductor with strong visible-light absorption and a highly negative conduction band potential, suffers from fast charge recombination and a tendency to aggregate during synthesis and operation^9,10^. Unlike CoFe_2_O_4_, CdO also lacks magnetic properties, which complicates its separation from treated water and limits practical application.

These complementary drawbacks highlight the need for a rational heterojunction design. In such a system, CoFe_2_O_4_ provides structural stability and magnetic recoverability, while CdO contributes strong visible-light response and enhanced reductive capability. The coupling of CoFe_2_O_4_ and CdO therefore offers a synergistic photocatalytic system. More importantly, appropriate band alignment enables the formation of an S-scheme heterojunction, which preserves highly energetic charge carriers while suppressing undesired electron–hole recombination. This configuration is particularly advantageous for dual-function photocatalysis, where simultaneous oxidative degradation of organic pollutants and reductive removal of metal ions are required.

To further improve dispersion, interfacial contact, and structural stability, bentonite was used as a layered support matrix^11^. Bentonite suppresses nanoparticle agglomeration and provides abundant surface functional groups and adsorption sites, which facilitate pollutant enrichment near reactive interfaces^12,13^. In addition, a green synthesis route using plant extracts was adopted to provide an environmentally friendly fabrication method while also enhancing surface functionalization and interfacial interactions.

Based on these considerations, this study reports the synthesis of a novel magnetic CF-CdO@B heterojunction photocatalyst via a combined co-precipitation and green synthesis strategy. The photocatalytic performance of the material is systematically evaluated for the simultaneous reduction of Cr(VI) and degradation of cefixime under natural sunlight irradiation. Detailed structural, optical, electrochemical, and mechanistic analyses are conducted to clarify the role of heterojunction formation and charge transfer pathways, while recyclability tests demonstrate the practical applicability of the catalyst for sustainable wastewater treatment.

## Experimental section

### Synthesis of catalyst

#### *Synthesis of CoFe*_*2*_*O*_*4*_* nanoparticles*

Cobalt ferrite (CoFe_2_O_4_) nanoparticles were synthesized via a co-precipitation method. First, an aqueous solution of cobalt nitrate hexahydrate [Co(NO_3_)_2_·6H_2_O, 0.1 M, 25 mL] was mixed with an aqueous solution of iron(III) nitrate nonahydrate [Fe(NO_3_)_3_·9H_2_O, 0.2 M, 25 mL]. The mixture was magnetically stirred at room temperature for 30 min to ensure complete homogenization. Subsequently, the pH was gradually adjusted to above 12 by the dropwise addition of sodium hydroxide solution under continuous stirring. The resulting suspension was heated to 80 °C and maintained under reflux for 2 h to facilitate the formation of CoFe_2_O_4_ precipitates. After completion of the reaction, the mixture was allowed to cool naturally to room temperature. Due to the magnetic nature of CoFe_2_O_4_, the precipitates were easily separated from the reaction medium using an external magnet. The collected solids were washed several times with deionized water and ethanol to remove residual ions and impurities. Finally, the product was dried at 60 °C and calcined at 550 °C for 2 h to improve crystallinity and magnetic properties.

#### Green synthesis of CdO nanoparticles

Fresh *Petroselinum crispum* leaves were collected from a local market in Birjand, Iran. The leaves were separated from the stems, thoroughly washed several times with distilled water to remove surface contaminants, and shade-dried at ambient conditions for two weeks. The dried leaves were then ground into a fine powder. For the preparation of the aqueous extract, 10 g of the powdered plant material was dispersed in 100 mL of double-distilled water and refluxed for 20 min. The mixture was then filtered to obtain a clear extract, which was used immediately for the green synthesis of nanoparticles.

Cadmium oxide (CdO) nanoparticles were synthesized via a green precipitation method using the *P. crispum* extract. Briefly, 50 mL of the plant extract was added dropwise to 25 mL of a 0.1 M aqueous solution of cadmium nitrate tetrahydrate under continuous magnetic stirring. The reaction mixture was maintained at 80 °C and stirred for 2 h, leading to the formation of a precipitate. After cooling to room temperature, the precipitate was collected by centrifugation and washed several times with double-distilled water to remove residual impurities. The product was then dried overnight at room temperature. Finally, the dried precursor was calcined at 500 °C for 2 h in a muffle furnace to obtain CdO nanoparticles.

#### Synthesis of CF-CdO@B heterojunction

The CF-CdO@B heterojunction catalyst was fabricated via a simple in situ assembly strategy by integrating the as-prepared CoFe_2_O_4_ and CdO components onto bentonite as a layered support. First, the synthesized CoFe_2_O_4_ nanoparticles were redispersed in deionized water under magnetic stirring to obtain a homogeneous suspension (solution A). In a separate vessel, a CdO precursor suspension derived from the Petroselinum crispum extract was prepared, denoted as solution B. Solutions A and B were then mixed, followed by the addition of bentonite (0.5 g) under continuous stirring at room temperature. The mixture was further stirred for 30 min to ensure uniform dispersion of the metal oxide nanoparticles and effective interfacial contact with the bentonite layers. During this process, CdO nanoparticles were anchored onto the bentonite surface while simultaneously establishing intimate interfacial interactions with CoFe_2_O_4_, leading to the formation of a heterojunction structure. After mixing, the solid product was separated and washed several times with deionized water and ethanol to remove physically adsorbed species and residual organic compounds from the plant extract. The obtained material was dried at room temperature and subsequently calcined at 500 °C for 2 h in a muffle furnace to improve crystallinity, enhance interfacial coupling among CoFe_2_O_4_, CdO, and bentonite, and yield the final CF-CdO@B heterojunction catalyst.

#### Evaluation of photocatalytic performance

The photocatalytic performance of the prepared catalysts was evaluated through the photoreduction of Cr(VI) and the photodegradation of cefixime under natural sunlight. The experiments were carried out during the summer of 2024 on cloudless days between 10:00 and 14:00, when the average solar irradiance was approximately 250 kLux. In a typical experiment, a known amount of photocatalyst was dispersed in 50 mL of an aqueous pollutant solution with a predefined initial concentration. The pH of the solution was adjusted to the desired value using dilute HCl or NaOH solutions. Prior to irradiation, the suspension was stirred in the dark for 30 min to achieve adsorption–desorption equilibrium between the catalyst and pollutant species. The photocatalytic reaction was then initiated by exposing the system to direct sunlight. At specific time intervals, 2 mL aliquots were withdrawn and the photocatalyst was rapidly separated by magnetic decantation. The residual concentration of Cr(VI) was determined by UV–Vis spectrophotometry using a standard colorimetric method, while the concentration of cefixime was measured from its characteristic absorption peak using the same technique. The removal efficiency and reaction kinetics were calculated using the following equations^14^:

$$\mathrm{Degradation}\, \mathrm{efficency}\, {\%}=\frac{{\mathrm{C}}_{0}-\mathrm{C}}{{\mathrm{C}}_{0}} \times 100$$

$$-\mathrm{ln}\left(\frac{\mathrm{C}}{{\mathrm{C}}_{0}}\right)=\mathrm{k}\mathrm{t}$$

Here, ${\mathrm{C}}_{0}$ (mg/L) is the initial concentration of the pollutant (Cr(VI) or cefixime), C (mg/L) is the concentration at irradiation time ttt, and k (min^−1^) is the apparent pseudo-first-order rate constant.

## Results and discussions

### Structure and morphology

**Fig. 1:**
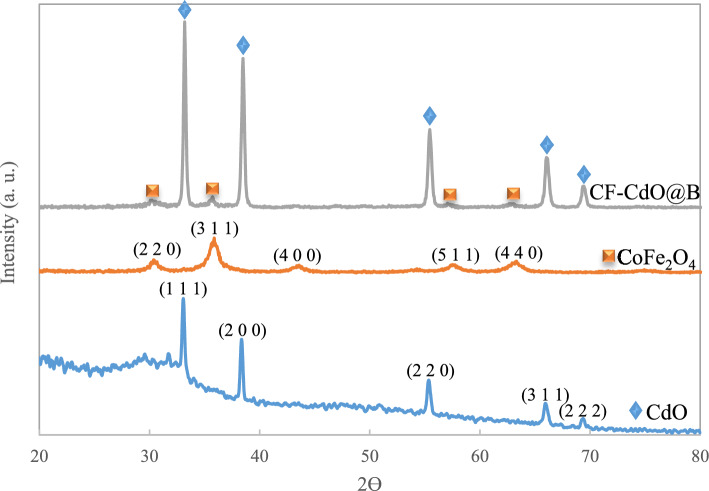
XRD patterns of samples.

X-ray diffraction (XRD) analysis, as shown in Fig. 1, was used to determine the crystal structure, phase composition, and crystallite size of the samples. The CoFe_2_O_4_ spectrum exhibited characteristic diffraction peaks at 30.62^°^, 36.24^°^, 43.37^°^, 57.92^°^, and 63.37^°^, which are in good agreement with JCPDS card no. 01–1121^15^. Similarly, the XRD pattern of CdO nanoparticles showed distinct peaks at 33.27^°^, 38.39^°^, 55.29^°^, 66.06^°^, and 69.46^°^, consistent with JCPDS card no. 075–0593^16^. In the CF-CdO@B composite, diffraction peaks corresponding to both CoFe_2_O_4_ and CdO were observed without significant peak shifts, confirming the successful incorporation of crystalline nanoparticles onto the bentonite support. In addition, no distinct diffraction peaks of bentonite were detected in the composite pattern, which can be attributed to its low loading content and the high dispersion of nanoparticles on its surface^12^.

**Fig. 2:**
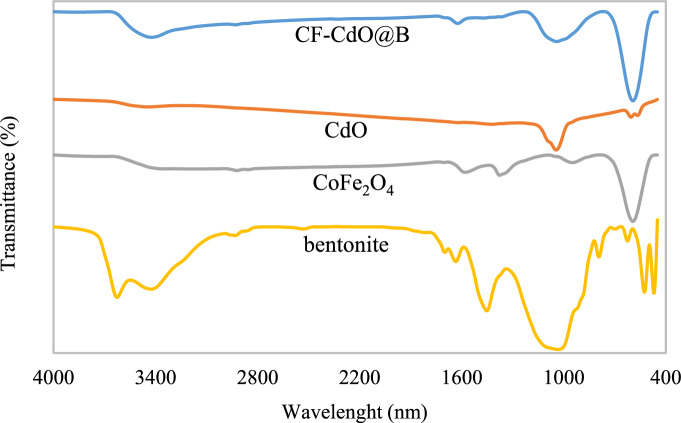
FTIR spectra of samples.

FT-IR spectroscopy in the range of 400–4000 cm^-1^ was used to identify the functional groups present on the surfaces of the samples. As shown in Fig. 2, a broad absorption band at 3100–3600 cm^-1^ is attributed to O–H stretching vibrations^17,18^. In addition, a weak band at 1618 cm^-1^ corresponds to the bending vibration of O–H groups^19^. These surface hydroxyl groups are important in the photocatalytic process, as they can react with photogenerated holes to form highly reactive ·OH radicals. Since CdO nanoparticles were synthesized using plant extract, characteristic bands related to C-O groups are also expected. In the FT-IR spectrum of CdO, the band around 1030 cm^-1^ is assigned to C-O stretching vibrations^20^, while the band at 550–600 cm^-1^ corresponds to Cd–O bonding vibrations^21^. For CoFe_2_O_4_, the absorption band at 589 cm^-1^ is attributed to Co–O and Fe–O stretching vibrations^22^. In the CF-CdO@B composite, a strong band at around 1000 cm^-1^ is assigned to Si–O-Si stretching vibrations of bentonite^23^. Although bentonite was not clearly detected in the XRD pattern due to its low content and high dispersion, its presence is confirmed by FT-IR analysis. Overall, the appearance of all expected characteristic bands in the CF-CdO@B spectrum confirms the successful synthesis and effective integration of CdO and CoFe_2_O_4_ onto the bentonite surface.

**Fig. 3:**
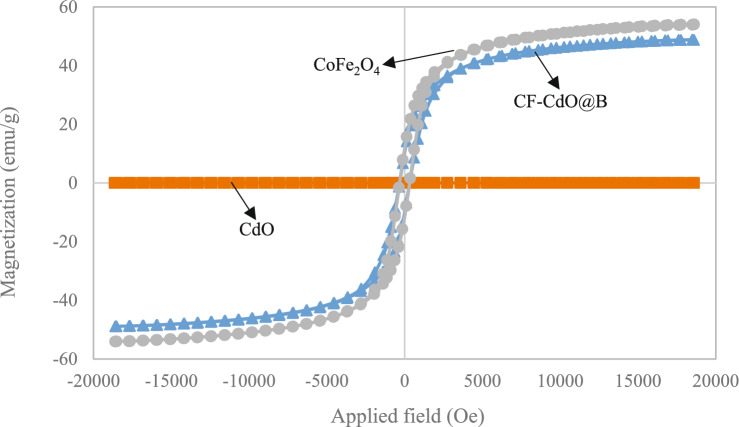
Magnetic hysteresis curves of samples.

The magnetic properties of the synthesized samples were evaluated using magnetization measurements. As shown in Fig. 3, CdO exhibits non-magnetic behavior^24^, whereas CoFe_2_O_4_ and CF-CdO@B display superparamagnetic characteristics with saturation magnetization values of 54.04 and 48.85 emu g^-1^, respectively. Despite the non-magnetic nature of CdO and bentonite, the CF-CdO@B composite retains a high magnetic response. This behavior is mainly attributed to the dominant contribution of CoFe_2_O_4_ within the composite structure. The strong magnetization enables rapid separation of CF-CdO@B from the reaction medium using an external magnetic field, demonstrating its potential as a reusable catalyst and reducing the risk of secondary pollution.

**Fig. 4:**
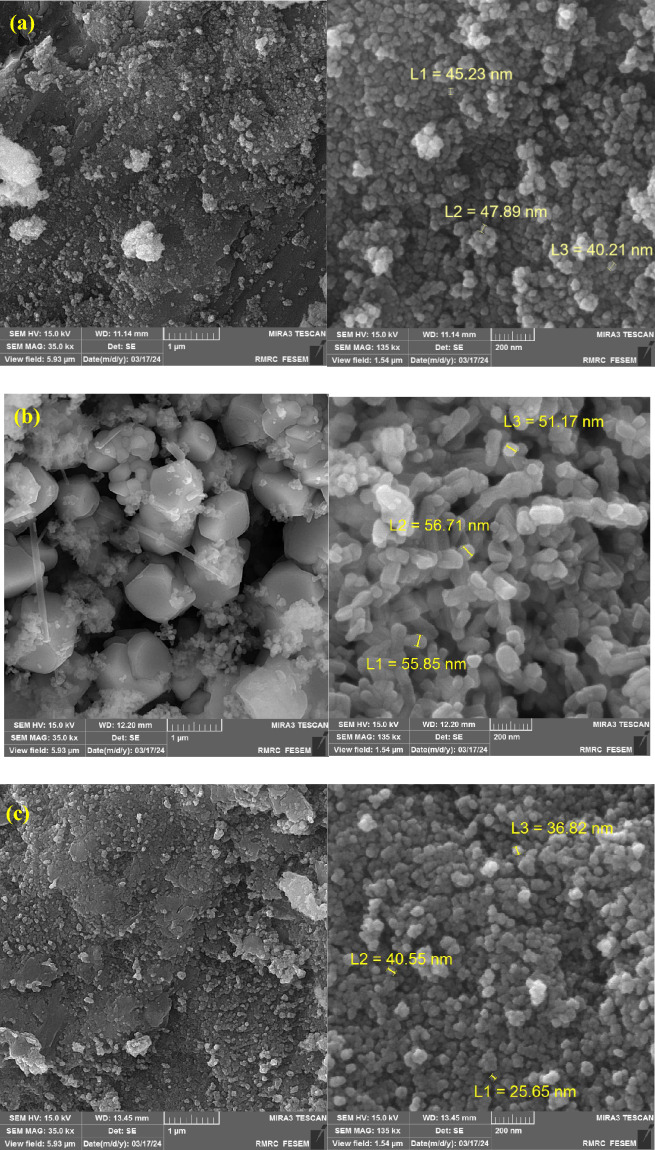
FESEM images of (**a**) CoFe_2_O_4_, (**b**) CdO and (**c**) CF-CdO@B.

FE-SEM analysis was performed to investigate the morphology of the synthesized samples. As shown in Fig. 4a and b, CdO and CoFe_2_O_4_ nanoparticles exhibit an approximately spherical morphology with some degree of aggregation. After immobilization on bentonite, the nanoparticles appear more uniformly dispersed with reduced agglomeration (Fig. 4c). This behavior can be attributed to the rapid anchoring of nanoparticles onto the bentonite surface during synthesis, which limits particle growth and suppresses aggregation. These results confirm that bentonite acts as an effective support for nanoparticle stabilization and dispersion. It should be noted that slight charging effects were observed during FE-SEM imaging due to the non-conductive nature of bentonite; however, these effects did not interfere with the clear identification of morphology and particle distribution.

**Fig. 5:**
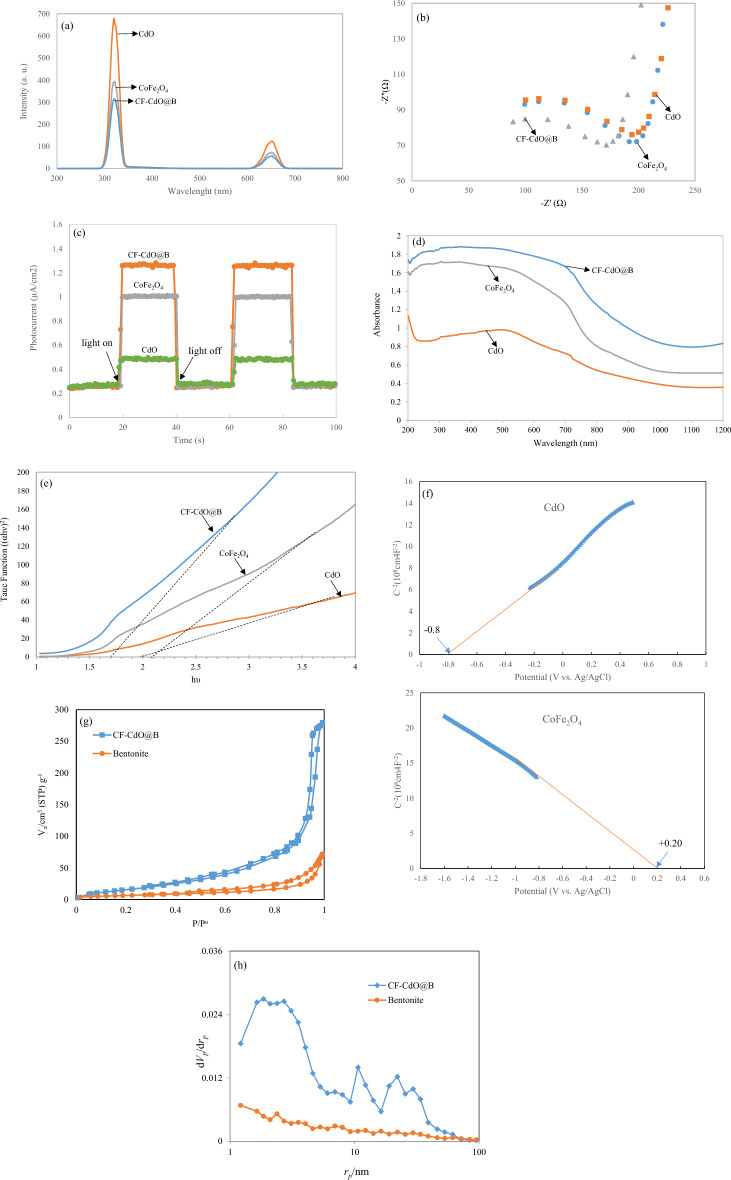
(**a**) PL, (**b**) EIS plots, (**c**) photocurrent response (**d**) UV–Vis DRS, (**e**) Tauc plots diagrams, and (**f**) Mott–Schottky curves, (**g**) N_2_ adsorption–desorption isotherms, (**h**) BJH pore size distribution curves of samples.

Photoluminescence (PL) spectroscopy was used to investigate charge carrier dynamics and defect-related recombination in the photocatalysts. PL provides insight into the radiative recombination of photogenerated electron–hole (e^-^/h^+^) pairs and is therefore an important indicator of charge separation efficiency. Upon excitation at 325 nm, the CF-CdO@B composite exhibited a lower emission intensity compared with pristine CoFe_2_O_4_ and CdO (Fig. 5a). This quenching of PL intensity indicates a significant reduction in charge carrier recombination. The behavior can be attributed to the formation of an effective heterojunction interface between CoFe_2_O_4_ and CdO, which facilitates interfacial charge transfer. Such an architecture promotes efficient migration of photogenerated charge carriers across the interface, thereby suppressing recombination losses and improving photocatalytic performance in pollutant degradation reactions.

Electrochemical impedance spectroscopy (EIS) was used to evaluate interfacial charge transfer behavior in the CF-CdO@B heterostructure. As shown in Fig. 5b, the Nyquist plot of CF-CdO@B exhibits a much smaller semicircle compared with pristine CoFe_2_O_4_ and CdO, indicating lower charge transfer resistance at the electrode–electrolyte interface. This improvement is attributed to the formation of an intimate heterointerface and strong electronic interaction between CoFe_2_O_4_ and CdO. These features facilitate more efficient charge transport and promote directional migration of photogenerated electrons and holes, while simultaneously suppressing interfacial recombination. Overall, the synergistic band alignment and enhanced charge separation endow CF-CdO@B with superior charge transport properties, which explain its improved photocatalytic performance compared with the individual components.

Photocurrent measurements were performed to further evaluate the charge separation efficiency of the CF-CdO@B heterostructure (Fig. 5c). Under intermittent light irradiation, CF-CdO@B exhibited a significantly higher and more stable photocurrent response compared with pristine CdO and CoFe_2_O_4_, indicating more efficient generation and transport of photogenerated charge carriers. The enhanced photocurrent density is attributed to the formation of a p-n heterojunction between n-type CdO and p-type CoFe_2_O_4_. This junction generates an internal electric field that promotes directional charge transfer and effectively suppresses electron–hole recombination. These results are consistent with the PL and EIS analyses and further confirm the improved charge separation efficiency, which is responsible for the enhanced photocatalytic performance of the composite.

The UV–Vis diffuse reflectance (DRS)^25^ spectra of pristine CdO, CoFe_2_O_4_, and the CF-CdO@B heterojunction nanocomposite demonstrate clear optical modifications induced by interfacial coupling (Fig. 5d). Compared with the individual components, the heterojunction shows enhanced absorption intensity and a noticeable red shift in the absorption edge, indicating improved light-harvesting ability in the visible region. The optical band gap energies were estimated from the Kubelka–Munk function and Tauc plots. The band gaps were calculated to be 2.0 eV for CdO and 2.2 eV for CoFe_2_O_4_, whereas the CF-CdO@B composite exhibits a reduced band gap of 1.7 eV (Fig. 5e). This band gap narrowing, together with the broadened absorption range, enhances photon utilization and promotes the generation of photogenerated charge carriers. These synergistic optical features contribute to the superior photocatalytic performance of the heterostructure under visible-light irradiation and emphasize the importance of band structure engineering in designing efficient photocatalysts.

Mott-Schottky (M-S) analysis was further conducted to determine the semiconductor type and flat-band potential of the individual components, providing complementary electrochemical insight into the heterojunction characteristics. As shown in Fig. 5f, the C^-2^-V plot of CdO exhibits a positive slope, confirming its n-type semiconducting nature with electrons as the majority charge carriers. The flat-band potential (E_fb_) derived from the x-intercept is approximately − 0.8 V vs. Ag/AgCl. In contrast, CoFe_2_O_4_ displays a negative slope, with an E_fb_ of approximately + 0.2 V vs. Ag/AgCl, indicating p-type semiconductor behavior dominated by hole carriers. The coexistence of n-type CdO and p-type CoFe_2_O_4_ within the CF-CdO@B composite strongly supports the formation of a well-defined heterointerface. The M-S analysis unambiguously identifies the complementary semiconductor characteristics of CdO and CoFe_2_O_4_, which constitute a solid foundation for establishing an internal electric field at the heterointerface-an essential prerequisite for efficient charge separation in the subsequent photocatalytic process.

While the M-S analysis provides insight into the electronic structure and interfacial charge transfer behavior, the surface textural properties were also evaluated to correlate morphology with photocatalytic activity. The nitrogen adsorption–desorption isotherms (Fig. 5f) of both bentonite and CF-CdO@B exhibit typical type IV behavior with a pronounced hysteresis loop at P/P₀ ≈ 0.4–1.0, confirming the mesoporous nature of the materials. Notably, CF-CdO@B shows a significantly higher nitrogen uptake over the entire relative pressure range, particularly at high P/P₀ (> 0.9), indicating the development of an expanded porous network with abundant accessible voids. Quantitative BET analysis further reveals a substantial enhancement in textural properties upon composite formation (Fig. 5g). The specific surface area increases from 23.733 m^2^ g^-1^ for raw bentonite to 94.73 m^2^ g^-1^ for CF-CdO@B, accompanied by a marked rise in total pore volume from 0.1049 to 0.4752 cm^3^ g^-1^. Additionally, the monolayer adsorption capacity (V_m_) increases from 4.4528 to 22.148 cm^3^ g^-1^, confirming the significant increase in available adsorption sites. This approximately fourfold enhancement in both surface area and pore volume highlights the critical role of nanoparticle immobilization in modifying the bentonite framework.

The BJH pore size distribution curves (Fig. 5h) provide further insight into the pore structure evolution. The raw bentonite exhibits a relatively narrow distribution centered within the mesoporous range (~ 2–5 nm) with low intensity, reflecting its limited pore accessibility. In contrast, CF-CdO@B displays a significantly broader and more intense pore size distribution, with a dominant peak in the 2–6 nm range and a noticeable presence of larger mesopores in the 10–30 nm region. Importantly, the pore volume distribution intensity (dV/dD) for CF-CdO@B is several times higher than that of bentonite (reaching ~ 0.024 compared to ~ 0.006), providing clear quantitative evidence for the generation of additional mesopores and interparticle voids.

This hierarchical mesoporous structure can be attributed to the effective dispersion of CoFe_2_O_4_ and CdO nanoparticles over the bentonite layers, which inhibits layer restacking and creates new pore channels. The strong agreement between BJH and BET results confirms that bentonite acts not merely as a passive support but as an active scaffold that promotes the formation of a highly porous architecture with enhanced surface accessibility. From a functional perspective, these improved textural properties play a decisive role in photocatalytic performance. The increased surface area and pore volume enhance the adsorption of cefixime and Cr(VI), while the hierarchical pore network facilitates rapid mass transfer and efficient diffusion of reactants. This structural advantage, combined with the improved charge separation demonstrated by PL and EIS analyses, accounts for the superior photocatalytic efficiency of CF-CdO@B.

The enhanced photocatalytic performance of CF-CdO@B can be further attributed to its nanoscale structural features. As confirmed by the BET analysis, the significant increase in specific surface area and pore volume leads to a higher density of accessible active sites and improved adsorption capacity for both cefixime and Cr(VI). The immobilization of CoFe_2_O_4_ and CdO nanoparticles onto bentonite inhibits aggregation and ensures uniform dispersion, which is crucial for maximizing interfacial contact. In addition, the hierarchical porous architecture facilitates rapid mass transfer and diffusion of reactant species toward active sites. More importantly, the intimate CdO/CoFe_2_O_4_ heterointerfaces at the nanoscale promote efficient spatial separation of photogenerated charge carriers, thereby suppressing recombination and enhancing redox reactions. These synergistic nanoscale effects collectively account for the superior photocatalytic activity of CF-CdO@B.

### Photocatalytic performance

#### Effect of pH on photocatalytic Cr(VI) reduction and cefixime degradation

**Fig. 6:**
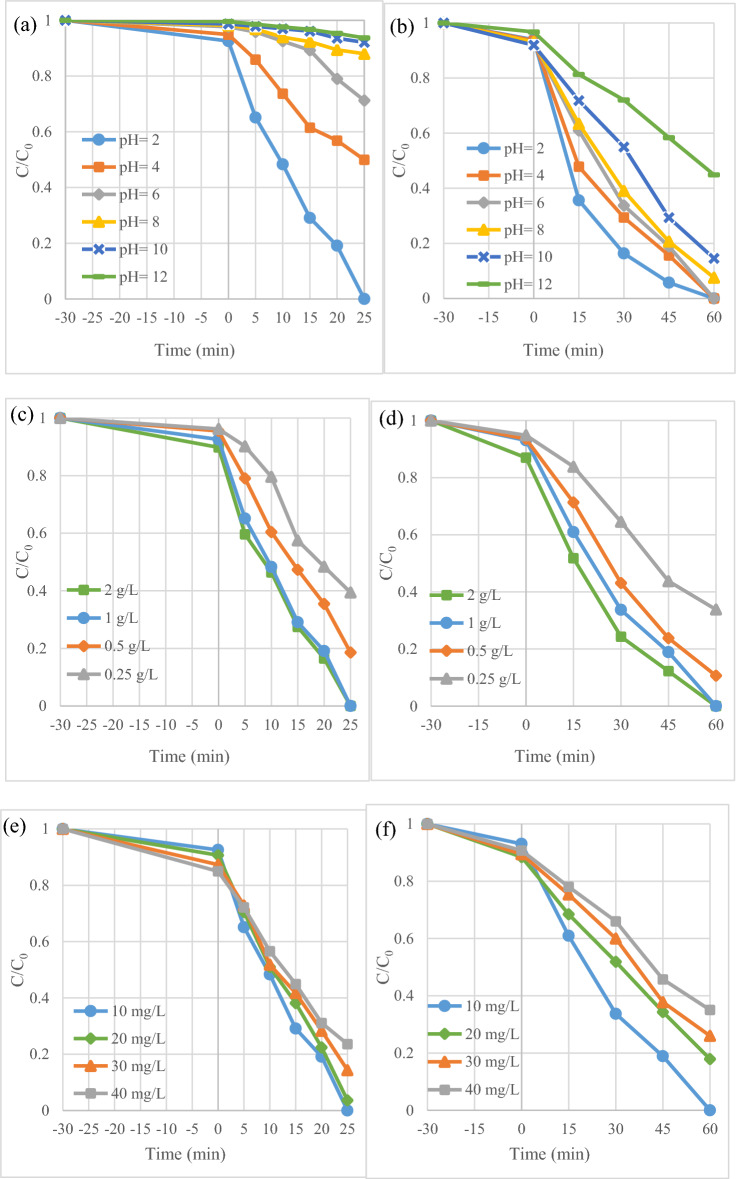

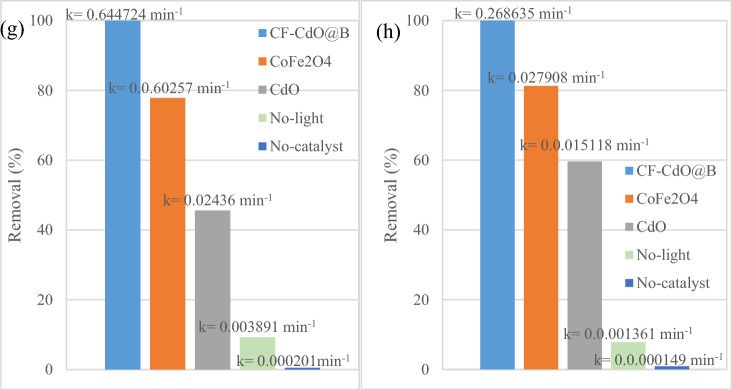
The impact of (**a**,**b**) initial solution pH, (**c**,**d**) dosage of photocatalyst, (**e**,**f**) pollutant concentrationon, and (**g**,**h**) photolysis, adsorption, photocatalysis, and type of photocatalyst on the reduction of Cr(VI) and degradation of cefixime over CF-CdO@B system.

The solution pH exerts a decisive influence on the photocatalytic performance of CF-CdO@B toward both Cr(VI) reduction and cefixime degradation, as illustrated in Fig. 6a and b). For Cr(VI), a rapid and highly pH-dependent reduction behavior is observed. Under acidic conditions, particularly at pH 2, almost complete reduction of Cr(VI) is achieved within 25 min of irradiation, whereas the reduction efficiency becomes negligible in alkaline media (pH 10–12). This pronounced enhancement at low pH is attributed to the favorable speciation of Cr(VI) as ${\mathrm{H}\mathrm{C}\mathrm{r}}_{2}{\mathrm{O}}_{7}^{-}$ and ${\mathrm{C}\mathrm{r}}_{2}{\mathrm{O}}_{7}^{2-}$, the abundance of protons facilitating electron-proton coupling, and the positively charged surface of CF-CdO@B, which strengthens electrostatic attraction toward negatively charged Cr(VI) species. In contrast, at higher pH values, ${\mathrm{C}\mathrm{r}\mathrm{O}}_{4}^{2-}$ dominates and the formation of Cr(OH)_3_ precipitates on the catalyst surface leads to active-site blockage, markedly suppressing the reduction kinetics^26^. Accordingly, pH 2 was selected as the optimal condition for Cr(VI) reduction.

For cefixime degradation, the photocatalytic activity of CF-CdO@B is less sensitive to pH variations compared with Cr(VI) reduction, yet remains highly effective over a wide acidic to near-neutral range. Complete degradation of cefixime is attained within 60 min at pH 2, 4, and 6, indicating that the photocatalyst retains strong oxidative capability under acidic to near-neutral conditions. Although acidic pH values yield marginally faster degradation rates, pH 6 was chosen as the optimal condition for cefixime degradation, as it provides full removal while offering greater environmental relevance and reduced chemical adjustment. When the pH is increased beyond neutral, the degradation efficiency gradually declines, reaching approximately 92%, 85%, and 55% at pH 8, 10, and 12, respectively, after 60 min. This decrease can be ascribed to diminished generation of reactive oxygen species, particularly ^•^OH radicals, and possible electrostatic repulsion between deprotonated cefixime molecules and the negatively charged catalyst surface in alkaline environments.

Overall, the distinct kinetic behaviors observed for Cr(VI) reduction and cefixime degradation highlight the dual functionality of CF-CdO@B. While Cr(VI) reduction is a fast process requiring strongly acidic conditions, cefixime degradation proceeds efficiently at near-neutral pH with a longer reaction time, underscoring the potential of this photocatalyst for simultaneous treatment of inorganic and organic contaminants under optimized conditions.

#### Effect of catalyst dosage on photocatalytic Cr(VI) reduction and cefixime degradation

The effect of CF-CdO@B dosage on the photocatalytic reduction of Cr(VI) and degradation of cefixime was evaluated under their respective optimal pH conditions (pH 2 for Cr(VI) and pH 6 for cefixime) with an initial pollutant concentration of 10 mg/L (Fig. 6c and d). Increasing the catalyst loading significantly enhances the removal efficiencies for both reactions. Complete Cr(VI) reduction and cefixime degradation were achieved at 1 and 2 g/L**,** after 25 min and 60 min, respectively, whereas lower dosages resulted in diminished performance. Specifically, Cr(VI) reduction dropped to 81% and 61% at 0.5 and 0.25 g/L, while cefixime degradation decreased to 89% and 66% under the same conditions. The enhanced performance at higher dosages is primarily due to the increased number of active sites available for pollutant adsorption and the generation of photogenerated electrons, holes, and reactive oxygen species, which accelerate both Cr(VI) reduction and cefixime oxidation. At low catalyst loadings, limited active sites and lower photon absorption reduce the formation of reactive species, leading to slower reaction rates. Notably, no significant improvement was observed when the dosage increased from 1 to 2 g/L, suggesting that 1 g/L is sufficient to achieve maximum photocatalytic activity, and further increases may cause light scattering or shielding effects that limit photon penetration. Therefore, 1 g/L was identified as the optimal catalyst dosage for both Cr(VI) reduction and cefixime degradation, representing a practical balance between catalytic efficiency and material economy. These results highlight the multifunctionality of CF-CdO@B, demonstrating its capability to efficiently remove both inorganic and organic pollutants under environmentally relevant and scalable conditions.

#### Effect of initial pollutant concentration on photocatalytic performance

The influence of initial pollutant concentration on the photocatalytic reduction of Cr(VI) and degradation of cefixime was investigated at concentrations ranging from 10 to 40 mg/L under their respective optimal pH conditions and using the optimized catalyst dosage (1 g/L). As illustrated in Fig. 6e and f, increasing the initial concentration led to a gradual decrease in removal efficiency and reaction rate for both pollutants. For Cr(VI), complete reduction was achieved within 25 min at 10 mg/L. However, when the concentration increased to 20, 30, and 40 mg/L, the final removal efficiencies declined progressively, indicating slower reaction kinetics at higher pollutant loadings. A similar trend was observed for cefixime degradation, where nearly complete removal was attained at 10 mg/L after 60 min, while increasing concentrations significantly reduced degradation efficiency. This behavior can be attributed to several interconnected factors. First, at higher pollutant concentrations, the number of pollutant molecules competing for the limited active sites on the catalyst surface increases, leading to surface saturation and reduced adsorption efficiency per molecule. Since photocatalytic reactions predominantly occur on or near the catalyst surface, this competitive adsorption directly limits the overall reaction rate. Second, increasing pollutant concentration reduces light penetration into the reaction medium due to enhanced solution opacity and light attenuation effects. This phenomenon decreases photon absorption by CF-CdO@B and consequently lowers the generation rate of photogenerated electrons, holes, and reactive oxygen species (ROS), which are responsible for Cr(VI) reduction and cefixime oxidation.

Moreover, at elevated concentrations, a larger amount of reactive species is required to convert pollutant molecules, while the production rate of ROS remains relatively constant under fixed irradiation and catalyst dosage. This imbalance between reactive species availability and pollutant molecules further contributes to the observed decrease in removal efficiency. Overall, the results confirm that CF-CdO@B exhibits high photocatalytic efficiency, particularly at environmentally relevant concentrations, while maintaining appreciable performance even at elevated pollutant loadings, highlighting its potential applicability in real wastewater treatment scenarios.

#### Control experiments and synergistic effect of CF-CdO@B

To further verify the photocatalytic nature of the processes and evaluate the contribution of individual components, comparative experiments were conducted for both Cr(VI) reduction and cefixime degradation under identical conditions (Fig. 6g and h). CF-CdO@B exhibited nearly complete removal efficiency (≈100%) for both pollutants, significantly outperforming the individual components. For Cr(VI) reduction, CoFe_2_O_4_ and CdO showed considerably lower efficiencies compared to the composite, while only ~ 10% reduction occurred under dark conditions and less than 2% in the absence of catalyst. Similarly, for cefixime degradation, CoFe_2_O_4_ and CdO achieved 81.26% and 59.63% removal, respectively, whereas only 7.84% degradation was observed without irradiation and 0.89% under direct photolysis. The negligible removal in dark conditions confirms that adsorption is not the dominant pathway, and the minimal photolysis efficiency excludes direct light-induced degradation as a major contributor. The inferior performance of CdO and CoFe_2_O_4_ alone can be attributed to limited charge separation efficiency and faster recombination of photogenerated electron–hole pairs. In contrast, the markedly enhanced activity of CF-CdO@B indicates that the integration of CoFe_2_O_4_ and CdO within the bentonite-supported structure improves the dispersion of active phases and facilitates interfacial charge transfer. This structural integration promotes more efficient utilization of photogenerated electrons for Cr(VI) reduction and enhances the formation of reactive oxygen species responsible for cefixime oxidation. Collectively, the results demonstrate that the superior photocatalytic performance originates from the synergistic interaction between the semiconductor components within the supported composite framework rather than from the isolated activity of each phase.

#### Kinetic analysis and synergistic effect evaluation

To further elucidate the photocatalytic performance and quantify the synergistic interactions within the CF-CdO@B heterojunction, the reaction kinetics of both Cr(VI) reduction and cefixime degradation were systematically analyzed under optimized conditions using a pseudo-first-order kinetic model^27–29^. For Cr(VI) reduction (pH 2, catalyst dosage 1 g/L, initial concentration 10 mg/L), the apparent rate constant (k) over CF-CdO@B was determined to be 0.6447 min^-1^, which is significantly higher than those obtained for the individual components, namely CoFe_2_O_4_ (0.0603 min^-1^) and CdO (0.0244 min^-1^). This remarkable enhancement clearly demonstrates the strong synergistic effect arising from heterojunction formation. The rate constant of the composite is more than an order of magnitude higher than that of the pristine materials, confirming that the interfacial charge transfer between CoFe_2_O_4_ and CdO effectively suppresses electron–hole recombination and accelerates the reduction process. In contrast, negligible kinetic activity was observed under dark conditions (0.0039 min^-1^) and in the absence of CF-CdO@B (0.0002 min^-1^), highlighting that the process is predominantly driven by photocatalytic reactions rather than adsorption or photolysis.

A similar kinetic trend was observed for cefixime degradation under optimized conditions (pH 6, catalyst dosage 1 g/L, initial concentration 10 mg/L). The CF-CdO@B photocatalyst exhibited a high apparent rate constant of 0.2686 min^-1^, which is substantially greater than those of CoFe_2_O_4_ (0.0279 min^-1^) and CdO (0.0151 min^-1^). The enhanced kinetic performance can be attributed to the efficient generation of reactive oxygen species facilitated by improved charge separation within the heterojunction structure. As in the case of Cr(VI), the reaction rates under dark conditions (0.00136 min^-1^) and without catalyst (0.00015 min^-1^) were negligible, confirming that photocatalysis is the dominant pathway. The significantly higher rate constants observed for CF-CdO@B in both oxidative (cefixime degradation) and reductive (Cr(VI) reduction) pathways provide strong kinetic evidence for its dual-function photocatalytic capability. Notably, the faster kinetics observed for Cr(VI) reduction compared to cefixime degradation can be attributed to the more favorable redox potential and rapid electron transfer processes under acidic conditions.

In addition to the pseudo-first-order kinetic model used for apparent rate constant determination, the photocatalytic behavior can be qualitatively interpreted using the Langmuir–Hinshelwood (L–H) mechanism^30,31^. In this model, pollutant molecules are first adsorbed onto the catalyst surface, followed by reaction with photogenerated reactive species, indicating that both adsorption–desorption equilibrium and interfacial charge transfer govern the overall reaction rate. The decrease in reaction rate at higher initial pollutant concentrations can be attributed to the saturation of active sites and the attenuation of light penetration, which collectively limit the availability of reactive species and accessible surface sites. Overall, the kinetic analysis confirms the superior photocatalytic performance of CF-CdO@B and highlights the synergistic contribution of CoFe_2_O_4_ and CdO, supporting its efficiency for simultaneous removal of organic and inorganic pollutants.

#### Radical scavenger studies

To identify the dominant reactive species involved in the photocatalytic processes and to gain deeper insight into the charge transfer mechanism, a series of radical scavenger experiments were conducted under optimal conditions for both Cr(VI) reduction and cefixime degradation. Silver nitrate (AgNO_3_), sodium oxalate (Na_2_C_2_O_4_), isopropyl alcohol (IPA), and benzoquinone (BQ) were employed as scavengers for electrons (e^-^), holes (h^+^), hydroxyl radicals (·OH), and superoxide radicals ($O_{2}^{ \cdot - }$), respectively. For Cr(VI) reduction, the photocatalytic efficiency decreased significantly from 100% (without scavenger) to 35.98% in the presence of AgNO_3_, indicating that photogenerated electrons play a dominant role in the reduction process. In contrast, the addition of sodium oxalate and isopropyl alcohol only slightly affected the efficiency (95.24% and 89.65%, respectively), suggesting that holes and ·OH radicals have negligible contributions. The presence of benzoquinone moderately suppressed the reduction efficiency to 68.37%, implying a secondary role of $O_{2}^{ \cdot - }$ radicals. These results clearly demonstrate that Cr(VI) reduction is primarily driven by highly reducing electrons.

For cefixime degradation, a different trend was observed. The degradation efficiency decreased markedly to 40.41% upon the addition of sodium oxalate, confirming that holes are the main oxidative species. The presence of isopropyl alcohol also reduced the efficiency to 53.24%, indicating a significant contribution from OH radicals. Meanwhile, benzoquinone caused a moderate decrease (71.61%), suggesting a supporting role of $O_{2}^{ \cdot - }$ radicals. In contrast, AgNO_3_ had a relatively minor effect (91.22%), indicating that electrons are not the primary reactive species in this process. Therefore, cefixime degradation is mainly governed by oxidative species, particularly h^+^ and ·OH radicals.

Importantly, the simultaneous presence of strong oxidative species (h⁺/·OH) and highly reductive electrons in the system provides compelling evidence against a conventional type-II heterojunction mechanism, in which the redox potentials would be weakened due to spatial charge separation. Instead, the preservation of highly energetic electrons (responsible for Cr(VI) reduction) and highly oxidative holes (responsible for cefixime degradation) strongly supports an S-scheme charge transfer pathway. In this mechanism, the recombination of low-energy charge carriers at the interface allows the retention of electrons in the conduction band of CdO and holes in the valence band of CoFe_2_O_4_, thereby maintaining strong redox ability. These findings are fully consistent with the band structure analysis and further confirm the proposed S-scheme photocatalytic mechanism.

#### Mechanistic insights into sunlight-induced photocatalysis by CF-CdO@B

The photocatalytic mechanism of CF-CdO@B under sunlight irradiation was further investigated. For this purpose, Mulliken’s electronegativity theory was employed to estimate the conduction band (E_CB_) and valence band (E_VB_) positions of the semiconductor components^32^.

1 $${\mathrm{E}}_{\mathrm{C}\mathrm{B}}=\upchi - {\mathrm{E}}_{\mathrm{C}}-0.5{\mathrm{E}}_{\mathrm{g}}$$

2 $${\mathrm{E}}_{\mathrm{V}\mathrm{B}}={\mathrm{E}}_{\mathrm{C}\mathrm{B}}+ {\mathrm{E}}_{\mathrm{g}}$$

Here, ${\mathrm{E}}_{\mathrm{C}}$ represents the energy of free electrons on the hydrogen scale (4.5 eV vs. NHE), and χ denotes the absolute electronegativity of the semiconductor. The χ values obtained from the literature were 5.8 eV for CoFe_2_O_4_^33^ and 4.67 eV for CdO^34^, which were used to determine the band edge positions. Accordingly, the E_CB_ values were estimated to be + 0.20 eV for CoFe_2_O_4_ and − 0.83 eV for CdO, while the corresponding E_VB_ values were calculated as + 2.40 eV and + 1.17 eV, respectively.

**Fig. 7:**
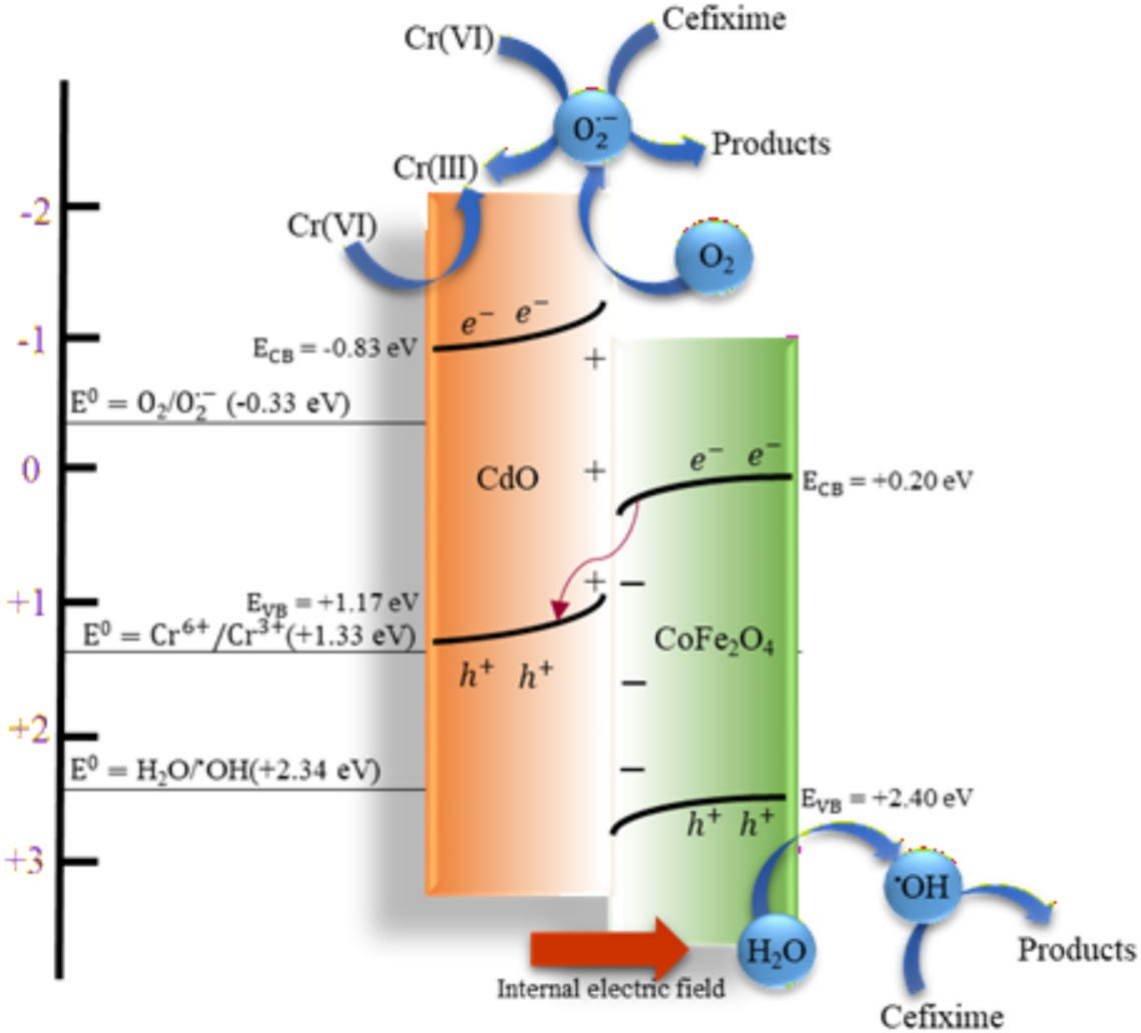
Mechanistic depiction of the S-scheme charge transfer in CF-CdO@B under sunlight.

Importantly, these theoretically derived band positions were experimentally verified by Mott-Schottky (M-S) analysis. As shown in Fig. 5f, CdO exhibits a positive slope, confirming its n-type character, whereas CoFe_2_O_4_ shows a negative slope, indicating p-type behavior. The flat-band potentials derived from the x-intercepts (approximately − 0.8 V vs. Ag/AgCl for CdO and + 0.2 V vs. Ag/AgCl for CoFe_2_O_4_) are in excellent agreement with the calculated band edge positions, providing direct electrochemical evidence for the proposed band alignment (Fig. 7). The difference in flat-band potentials further implies a Fermi level offset between the two semiconductors, which is responsible for the formation of an internal electric field upon contact. Building upon this experimentally validated band structure, the charge transfer mechanism for cefixime degradation and Cr(VI) reduction under sunlight irradiation is schematically illustrated in Fig. 7. Upon light excitation, electrons in both CdO and CoFe_2_O_4_ are promoted from the valence band to the conduction band. Although a conventional type-II heterojunction would predict electron transfer from CB(CdO) to CB(CoFe_2_O_4_) and hole migration from VB(CoFe_2_O_4_) to VB(CdO), such a pathway is inconsistent with the significantly enhanced photocatalytic activity of the heterostructure compared to its individual components, as well as PL, EIS and photocurrent response. More importantly, the radical scavenger experiments provide direct experimental evidence against this scenario. The pronounced suppression of Cr(VI) reduction in the presence of electron scavenger (AgNO_3_), together with the strong inhibition of cefixime degradation by hole and ·OH scavengers, clearly demonstrates the simultaneous participation of highly reductive electrons and highly oxidative species, confirming the preservation of strong redox potentials. Therefore, the photocatalytic system follows an S-scheme charge transfer mechanism. Due to the Fermi level difference between CoFe_2_O_4_ and CdO, electrons spontaneously migrate from CB(CoFe_2_O_4_) to VB(CdO) upon contact until equilibrium is established, generating a built-in electric field at the interface. This internal field induces band bending and drives the selective recombination of low-energy electrons in CB(CoFe_2_O_4_) with low-energy holes in VB(CdO), while preserving high-energy charge carriers—namely, strongly reducing electrons in CB(CdO) (− 0.83 eV) and strongly oxidizing holes in VB(CoFe_2_O_4_) (+ 2.40 eV). Consequently, the retained holes in VB(CoFe_2_O_4_) oxidize water molecules to generate ·OH radicals (E⁰( H_2_O/·OH) =  + 2.34 V vs. NHE)^29^, whereas the retained electrons in CB(CdO) reduce dissolved O_2_ to ${\mathrm{O}}_{2}^{\cdot -}$ radicals (E⁰(${\mathrm{O}}_{2}$/${\mathrm{O}}_{2}^{\cdot -}$) =  − 0.33 V vs. NHE)^35^ and simultaneously reduce Cr(VI) to Cr(III) (E⁰(${\mathrm{C}\mathrm{r}}^{6+}$/${\mathrm{C}\mathrm{r}}^{3+}$) =  + 1.33 V vs. NHE)^36^. The synergistic effect of the internal electric field and the high surface area of the bentonite support significantly enhances charge separation, suppresses recombination, and stabilizes the heterostructure, ultimately governing the superior dual-function photocatalytic performance of CF-CdO@B.

#### Stability and reusability of the magnetic photocatalyst

**Fig. 8:**
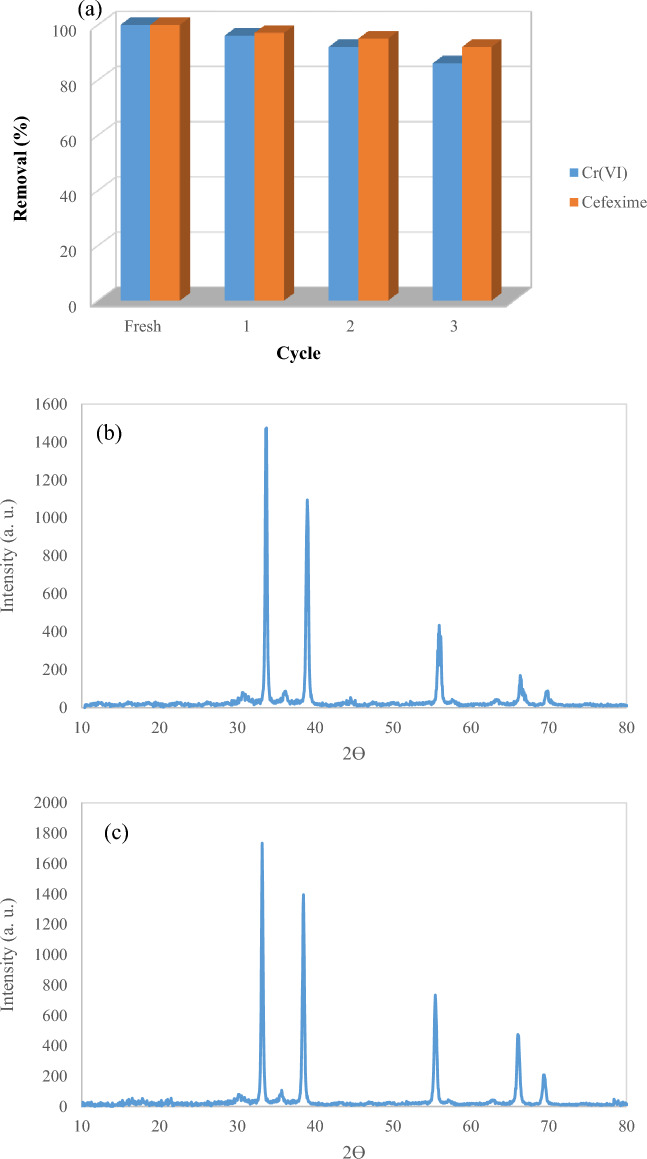
(**a**) Stability and reusability of CF-CdO@B during repeated cycles of cefixime degradation and Cr(VI) reduction and XRD patterns of used CF-CdO@B after photocatalytic cycles for (**b**) Cr(VI) reduction and (**c**) cefixime degradation.

Stability and reusability are critical factors for the practical application of photocatalysts. Therefore, recycling experiments were conducted to evaluate the stability of CF-CdO@B for both cefixime degradation and Cr(VI) reduction. After each photocatalytic run under optimal conditions, the catalyst was readily separated from the reaction medium using an external magnetic field and reused in subsequent cycles. As illustrated in Fig. 8a, after three consecutive runs, the degradation efficiency of cefixime decreased slightly from 100 to 92%, while the reduction efficiency of Cr(VI) declined from 100 to 86%, indicating good recyclability of the catalyst. To further assess chemical stability, Inductively Coupled Plasma Optical Emission Spectroscopy (ICP-OES) was performed after three cycles. The results revealed that metal ion leaching depends on the reaction conditions. Under acidic conditions (Cr(VI) reduction), the cumulative leaching of Cd and Co/Fe was approximately 7% and 5%, respectively. In contrast, lower leaching values of about 4% (Cd) and 3% (Co/Fe) were observed under near-neutral conditions (cefixime degradation). This difference can be attributed to the enhanced dissolution of metal oxides in acidic media. To further verify the structural stability of the catalyst, XRD analysis was conducted on the spent CF-CdO@B after three consecutive photocatalytic cycles (Cr(VI) reduction and cefixime degradation). As shown in Fig. 8b and c, the XRD patterns of the reused catalyst show no significant shift in peak positions, no loss of crystallinity, and no appearance of new phases compared to the fresh sample. The characteristic diffraction peaks of both CoFe_2_O_4_ and CdO remain clearly observable, confirming that the crystal structure of the composite is well preserved during photocatalytic operation. This indicates that the bulk framework of CF-CdO@B remains stable, and the slight decline in activity is mainly associated with minor surface metal leaching rather than structural degradation. Despite this moderate leaching, the photocatalyst retained high activity after repeated use, confirming its acceptable stability, magnetic recoverability, and reusability. The observed leaching levels are also consistent with previously reported studies, where partial metal dissolution under acidic photocatalytic conditions is commonly observed.

#### Photocatalytic performance in real water matrices

Considering the complexity and variability of real water matrices, the practical applicability of the CF-CdO@B photocatalyst was evaluated using different real water sources. For cefixime degradation, experiments were conducted in tap water, river water, and hospital wastewater under optimized conditions (pH 6, catalyst dosage 1 g/L, and cefixime concentration 10 mg/L). The photocatalyst exhibited high degradation efficiencies of 94%, 91%, and 85%, respectively, compared to complete degradation (100%) in distilled water. The slight decrease in performance in real water matrices can be attributed to the presence of coexisting inorganic ions (e.g., Cl^-^, SO_4_^2-^, NO_3_^-^, Ca^2+^, and Mg^2+^) as well as natural organic matter and dissolved organic carbon (DOC/TOC). These species may competitively adsorb on the catalyst surface, reducing the number of available active sites, and can also act as scavengers for reactive oxygen species (·OH and $O_{2}^{ \cdot - }$), thereby partially inhibiting the photocatalytic reaction. Despite these interference effects, only a moderate reduction in efficiency was observed, confirming the strong anti-interference capability and robustness of CF-CdO@B in complex water matrices. In contrast, the reduction of Cr(VI) was examined using tap water, river water, and wastewater from a leather processing facility under strongly acidic conditions. Complete reduction (≈100%) was consistently achieved in all tested matrices. This behavior can be attributed to the highly favorable thermodynamics and rapid kinetics of Cr(VI) reduction under acidic conditions, where the strong reductive potential of photogenerated electrons in the CF-CdO@B system dominates the process, and the influence of coexisting ions and organic matter becomes negligible due to the fast electron transfer rate and strong driving force of the reaction.

#### Comparative performance of CF-CdO@B with reported photocatalysts

The comparative analysis presented in Table 1 clearly demonstrates that the CF-CdO@B photocatalyst developed in this work exhibits superior photocatalytic performance compared to most previously reported systems. Under natural sunlight irradiation, the composite achieves complete removal of Cr(VI) within 25 min and cefixime within 60 min, corresponding to removal efficiencies of approximately ~ 100% for both pollutants. In contrast, many of the reported photocatalysts require significantly longer reaction times (typically 60–180 min or more) and often depend on artificial light sources such as Xe or UV lamps to reach comparable efficiencies. In particular, the CF-CdO@B system consistently outperforms a wide range of state-of-the-art materials, including semiconductor heterojunctions, metal–organic frameworks, and doped metal oxides, which generally exhibit lower efficiencies (typically 60–99%) under similar or more demanding irradiation conditions. The exceptional performance of CF-CdO@B can be attributed to the synergistic effects of its S-scheme heterojunction architecture, enhanced charge carrier separation, improved adsorption capacity provided by bentonite, and efficient utilization of natural sunlight. Moreover, the magnetic nature of the composite ensures facile recovery without compromising its activity over repeated cycles. Overall, the comparative data confirm that CF-CdO@B is a highly efficient dual-functional photocatalyst, capable of achieving near-complete (≈100%) removal of both inorganic (Cr(VI)) and organic (cefexime) pollutants under environmentally sustainable conditions, making it highly competitive with and in many cases superior to previously reported photocatalytic systems.

**Table 1:** Comparative photocatalytic performance of CF-CdO@B with previously reported photocatalysts for Cr(VI) reduction and cefixime degradation under different light sources.

Catalyst	Pollutant	Source of stimulation	Time (min)	Efficiency (%)
CF-CdO@B	Cr(VI)	sunlight	25	~ 100[ This work]
Cu_2_O/g-C_3_N_4_ S-scheme	Cr(VI)	Xe lamp	120	77.7^37^
BITS	Cr(VI)	Xe lamp	50	100^38^
ZnxCd_1− x_In_2_S_4_	Cr(VI)	Xe lamp	60	99.5^39^
Bi_2_MoO_6_/Bi_2_S_3_	Cr(VI)	Xe lamp	120	88^40^
BiPO_4_/CuBi_2_O_4_	Cr(VI)	Xe lamp	90	60.3^41^
C-doped BiOCl/Bi_2_S_3_	Cr(VI)	Xe lamp	120	99.5^42^
UNiMOF/BiVO_4_/S-C_3_N_4_	Cr(VI)	Xe lamp	120	93.6^43^
g-C_3_N_4_	Cr(VI)	visible light	90	98.5^44^
NFO/BBWO/BOI	Cr(VI)	LED lamp	60	95^45^
WO_3_/In_2_S_3_	Cr(VI)	Xe lamp	60	98.5^46^
NUBA	Cr(VI)	Xe lamp	30	96^47^
BiVO_4_/FeVO_4_@rGO	Cr(VI)	Xe lamp	90	90.9^48^
Cu_2_O/g-C_3_N_4_	Cr(VI)	Xe lamp	120	77.7^37^
Zn_3_In_2_S_6_/Bi_2_O_3_	Cr(VI)	Xe lamp	120	99.67^49^
Ag_3_VO_4_/AgPMo	Cr(VI)	Xe lamp	150	94.8^27^
MIL-100(Fe)/PANI	Cr(VI)	Xe lamp	120	100^50^
NH_2_-UiO-66 (Zr)	Cr(VI)	Xe lamp	120	99.5^51^
SVGA	Cr(VI)	Xe lamp	120	100^52^
C–TiO_2_/Cd_0.5_Zn_0.5_S	Cr(VI)	Xe lamp	120	95.5^53^
ZnO/ZrO_2_	Cr(VI)	UV light	150	62^54^
Bi_2_Mo_2_O_9_	Cr(VI)	UV light	180	64^55^
ZnIn_2_S_4_/CeO_2_	Cr(VI)	Xe lamp	80	97.7^56^
CoF@N-CS@ZO	Cr(VI)	Xe lamp	85	96.9^57^
CuS	Cr(VI)	Visible lamp	168.20	98.54^58^
CF-CdO@B	Cr(VI)	sunlight	60	~ 100[ This work]
ZnO/a-Fe_2_O_3_	cefexime	UV–vis lamp	127	99.1^59^
MIL-53(Fe)/urchin-like g-C_3_N_4_	cefexime	Xe lamp	120	94^60^
BiFeO_3_/Magnetic	cefexime	LED lamp	32	91.8^61^
TiO_2_/GO/Chitosan	cefexime	UV lamp	60	95.34^30^
TiO_2_	cefexime	UV lamp	60	90^62^
Bi_12_TiO_20_	cefexime	UV lamp	180	94.93^63^
MIL 125(Ti) mixed linker/g-C_3_N_4_	cefexime	Xe lamp	120	98^64^
N-TiO_2_/GO/titan grid sheets	cefexime	LED lamp	240	80^65^
TiO_2_-NPs	cefexime	UV lamp	90	90^2^
zeolite/SnO_2_/CuO	cefexime	sunlight	150	89.65^66^
g-C_3_N_4_/Ce–ZnO/Ti	cefexime	Vis lamp	330	96^67^
ZnFe_2_O_4_	cefexime	sunlight	90	100^68^
Ag_2_S/BiVO_4_/AgI	cefexime	Xe lamp	70	95.31^69^
BiO_1-x_Br/BiOI_1-x_-CdS	cefexime	halogen lamp	120	95.8^70^
Bi_12_TiO_20_	cefexime	UV lamp	180	94.93^63^
MIL 125(Ti) mixed linker/g-C_3_N_4_	cefexime	Xe lamp	120	98^64^
N-TiO_2_/GO/titan grid sheets	cefexime	LED lamp	240	80^65^
TiO_2_-NPs	cefexime	UV lamp	90	90^2^
zeolite/SnO_2_/CuO	cefexime	sunlight	150	89.65^66^
g-C_3_N_4_/Ce–ZnO/Ti	cefexime	Vis lamp	330	96^67^
ZnFe_2_O_4_	cefexime	sunlight	90	100^68^
Ag_2_S/BiVO_4_/AgI	cefexime	Xe lamp	70	95.31^69^
BiO_1-x_Br/BiOI_1-x_-CdS	cefexime	halogen lamp	120	95.8^70^

## Conclusion

In this work, a magnetic CoFe_2_O_4_/CdO@bentonite S-scheme heterostructure was successfully designed and fabricated via a combination of co-precipitation and green synthesis strategies. The resulting photocatalyst exhibited excellent dual-function activity for the simultaneous reduction of Cr(VI) and degradation of cefixime under natural sunlight, outperforming its individual components. The enhanced photocatalytic performance is attributed to the formation of an efficient S-scheme heterojunction, which facilitates directional charge transfer, suppresses electron–hole recombination, and preserves strong redox capabilities. Kinetic analysis further confirmed significantly accelerated reaction rates, demonstrating the synergistic interaction between CoFe_2_O_4_ and CdO within the bentonite-supported structure. In addition, bentonite played an important role in improving nanoparticle dispersion, enhancing pollutant adsorption, and stabilizing the heterostructure. The photocatalyst also showed excellent magnetic recoverability, stability, and reusability, maintaining high efficiency over repeated cycles. Its applicability was further validated in real water matrices, where high removal efficiencies were maintained despite the presence of competing species. Overall, this study provides a rational strategy for constructing multifunctional S-scheme photocatalysts and offers an environmentally sustainable approach for the treatment of complex wastewater containing both inorganic and organic pollutants. The results also provide useful insights for the future development and scale-up of solar-driven photocatalytic systems for practical environmental applications.

## Data Availability

The authors declare that the data supporting the findings of this study are available in this published article. Should any raw data files be needed in another format they are available from the corresponding author upon reasonable request.
